# Supplementary material for: An Open One-Step RT-qPCR for SARS-CoV-2 detection
Source: PLoS One. 2024 Jan 25;19(1):e0297081. doi: 10.1371/journal.pone.0297081 (PMC10810446; doi:10.1371/journal.pone.0297081)
Supplement: S1 Table — (DOCX) [file pone.0297081.s005.docx]

**Supplemental Table 1. Dye-based RT-qPCR reaction mix using M-MLV RT and Pfu-Sso7d.**

| **One-Step RT-qPCR (M-MLV/Pfu)** | **Volume (μL)** | **Final Concentration** |
| --- | --- | --- |
| RNA sample | 5 | - |
| 10 μM Forward PCR primer | 0.6 | 300 nM |
| 10 μM Reverse PCR primer | 0.6 | 300 nM |
| 10 mM dNTPs | 0.8 | 400 nM |
| 5X Homemade Buffer | 4 | 1X |
| 100 mM DTT | 2 | 10 mM |
| 20X EvaGreen | 1 | 1X |
| Pfu Ss07d (0.6 mg/mL) | 0.5 | 15 ng/μL |
| M-MLV RT (0.02 mg/mL) | 0.125 | 0.13 ng/μL |
| Nuclease-Free Water | 5.375 | - |
| Total Reaction Volume | 20 |  |
