# Supplementary material for: An Open One-Step RT-qPCR for SARS-CoV-2 detection
Source: PLoS One. 2024 Jan 25;19(1):e0297081. doi: 10.1371/journal.pone.0297081 (PMC10810446; doi:10.1371/journal.pone.0297081)
Supplement: S2 Table — (DOCX) [file pone.0297081.s006.docx]

**Supplemental Table 2. Dye-based One-Step RT-qPCR cycling conditions.**

| **Step** | **Temperature (°C)** | **Duration** |
| --- | --- | --- |
| 1 | 50 | 15 min |
| 2 | 98 | 2 min |
| Repeat steps 3 and 4 by 40 cycles | | |
| 3 | 98 | 10 s |
| 4* | 55 | 10 s |
| Melt Curve | | |
| 5 | 95 | 15 s |
| 6 | 60 | 1 min |
| 7 | 95 | 15 s |

*In this step fluorescence signal acquisition occurs (EvaGreen)
