# Supplementary material for: An Open One-Step RT-qPCR for SARS-CoV-2 detection
Source: PLoS One. 2024 Jan 25;19(1):e0297081. doi: 10.1371/journal.pone.0297081 (PMC10810446; doi:10.1371/journal.pone.0297081)
Supplement: S3 Table — Comparative Cq data for the TaqPath One-Step RT-qPCR kit and the probe-based open RT-qPCR reaction mix. Assigned sample number (# Sample) and clinical sample identifier (ID) are displayed. The clinical reports of the samples before they were re-tested by the two kits are also indicated in parentheses. The sample whose report was altered is denoted in bold. (-): negative samples, (+): positive samples, ND: non-detected. (DOCX) [file pone.0297081.s007.docx]

**Supplemental Table 3. Comparative Cq data between a commercial RT-qPCR kit and an Open RT-qPCR method based on homebrew M-MLV RT and Taq DNA pol**.

|  | | **Commercial Kit** | | | | **Open RT-qPCR (M-MLV/Taq)** | | | |
| --- | --- | --- | --- | --- | --- | --- | --- | --- | --- |
| **# Sample** | **ID** | **N1** | **N2** | **RNAse P** | **Report** | **N1** | **N2** | **RNAse P** | **Report** |
| 1 | *1215-023* (+) | 13.28 | 14.01 | 26.42 | Positive | 14.53 | 14.97 | 26.08 | Positive |
| 2 | *1229-055* (+) | 14.40 | 14.08 | 28.31 | Positive | 15.97 | 16.47 | 28.09 | Positive |
| 3 | *1228-001* (+) | 18.35 | 19.51 | 27.17 | Positive | 19.43 | 20.22 | 27.64 | Positive |
| 4 | *1228-048* (+) | 18.46 | 17.95 | 25.41 | Positive | 19.36 | 18.26 | 26.31 | Positive |
| 5 | *1230-140* (+) | 18.78 | 18.97 | 25.66 | Positive | 19.68 | 19.1 | 29.15 | Positive |
| 6 | *1228-047* (+) | 19.57 | 19.54 | 30.80 | Positive | 17.67 | 18.29 | 28.00 | Positive |
| 7 | *1228-066* (+) | 19.98 | 19.76 | 25.05 | Positive | 20.49 | 19.97 | 25.35 | Positive |
| 8 | *1230-024* (+) | 20.27 | 20.62 | 28.46 | Positive | 20.72 | 21.42 | 27.13 | Positive |
| 9 | *1229-034* (+) | 20.41 | 20.91 | 31.95 | Positive | 20.72 | 20.76 | 31.40 | Positive |
| 10 | *1228-035* (+) | 21.05 | 20.20 | 31.81 | Positive | 21.88 | 22.64 | 33.91 | Positive |
| 11 | *1228-096* (+) | 23.55 | 23.29 | 25.71 | Positive | 25.75 | 23.52 | 25.70 | Positive |
| 12 | *1230-096* (+) | 23.60 | 23.12 | 27.37 | Positive | 23.58 | 23.72 | 26.47 | Positive |
| 13 | *1230-142* (+) | 24.20 | 24.84 | 31.77 | Positive | 25.38 | 25.76 | 30.61 | Positive |
| 14 | *1229-054* (+) | 24.24 | 24.35 | 27.38 | Positive | 24.88 | 26.46 | 27.68 | Positive |
| 15 | *1228-040* (+) | 25.73 | 25.39 | 27.73 | Positive | 26.82 | 25.83 | 27.95 | Positive |
| 16 | *1230-101* (+) | 26.10 | 25.91 | 30.24 | Positive | 27.94 | 26.28 | 33.47 | Positive |
| 17 | *1228-021* (+) | 26.42 | 26.25 | 30.22 | Positive | 27.03 | 27.35 | 30.14 | Positive |
| 18 | *1228-102* (+) | 31.50 | 31.49 | 26.74 | Positive | 32.89 | 34.61 | 26.33 | Positive |
| 19 | *1230-044* (+) | 32.14 | 31.84 | 29.17 | Positive | 34.78 | 32.34 | 29.24 | Positive |
| **20** | ***1229-033* (+)** | ND | ND | 29.52 | Negative | ND | ND | 29.59 | Negative |
| 21 | *0421-0P1* (-) | ND | ND | 25.29 | Negative | ND | ND | 26.90 | Negative |
| 22 | *0421-0P2* (-) | ND | ND | 26.73 | Negative | ND | ND | 26.56 | Negative |
| 23 | *0414-0P3* (-) | ND | ND | 28.39 | Negative | ND | ND | 29.70 | Negative |
| 24 | *0414-0P4* (-) | ND | ND | 27.72 | Negative | ND | ND | 26.91 | Negative |
| 25 | *0414-0P5* (-) | ND | ND | 27.57 | Negative | ND | ND | 27.60 | Negative |
| 26 | *0414-0P6* (-) | ND | ND | 28.29 | Negative | ND | ND | 27.08 | Negative |
| 27 | *0414-0P7* (-) | ND | ND | 26.98 | Negative | ND | ND | 26.87 | Negative |
| 28 | *0414-0P8 (-)* | ND | ND | 27.78 | Negative | ND | ND | 26.03 | Negative |
| 29 | *0414-0P9* (-) | ND | ND | 25.34 | Negative | ND | ND | 26.26 | Negative |
| 30 | *0414-P10* (-) | ND | ND | 29.45 | Negative | ND | ND | 28.64 | Negative |
| 31 | *0414-P11* (-) | ND | ND | 28.95 | Negative | ND | ND | 28.88 | Negative |
| 32 | *0414-P12* (-) | ND | ND | 26.98 | Negative | ND | ND | 27.07 | Negative |
| 33 | *0414-P13* (-) | ND | ND | 26.54 | Negative | ND | ND | 26.11 | Negative |
| 34 | 0414-P14 (-) | ND | ND | 25.16 | Negative | ND | ND | 26.56 | Negative |
| 35 | *0414-P15* (-) | ND | ND | 27.11 | Negative | ND | ND | 27.90 | Negative |
| 36 | *0414-P16* (-) | ND | ND | 29.74 | Negative | ND | ND | 28.50 | Negative |
| 37 | *0414-P17* (-) | ND | ND | 25.85 | Negative | ND | ND | 28.14 | Negative |
| 38 | *0414-P18* (-) | ND | ND | 27.74 | Negative | ND | ND | 29.20 | Negative |
| 39 | *0414-P19* (-) | ND | ND | 25.45 | Negative | ND | ND | 26.87 | Negative |
| 40 | *0414-P20* (-) | ND | ND | 28.67 | Negative | ND | ND | 27.45 | Negative |

Comparative Cq data for the TaqPath One-Step RT-qPCR kit and the probe-based open RT-qPCR reaction mix. Assigned sample number (# Sample) and clinical sample identifier (ID) are displayed. The clinical reports of the samples before they were re-tested by the two kits are also indicated in parentheses. The sample whose report was altered is denoted in bold. **(-):** negative samples, **(+):** positive samples, **ND:** non-detected.
