# Supplementary material for: An Open One-Step RT-qPCR for SARS-CoV-2 detection
Source: PLoS One. 2024 Jan 25;19(1):e0297081. doi: 10.1371/journal.pone.0297081 (PMC10810446; doi:10.1371/journal.pone.0297081)
Supplement: S4 Table — Comparative Cq data for the TaqPath One-Step RT-qPCR kit and the dye-based open RT-qPCR reaction mix. Assigned sample number (# Sample) and clinical sample identifier (ID) are displayed. The clinical reports of the samples before they were re-tested by the two kits are also indicated in parentheses. The samples whose reports were altered are denoted in bold. (-): negative samples, (+): positive samples, ND: non-detected. (DOCX) [file pone.0297081.s008.docx]

**Supplemental Table 4. Comparative Cq data between a commercial RT-qPCR kit and an Open RT-qPCR method based on homebrew M-MLV RT and Pfu-Sso7d.**

|  | | **Commercial Kit** | | | | **Open RT-qPCR (M-MLV/Pfu)** | | | |
| --- | --- | --- | --- | --- | --- | --- | --- | --- | --- |
| **# Sample** | **ID** | **N1** | **N2** | **RNAse P** | **Report** | **N1** | **N2** | **RNAse P** | **Report** |
| 1 | *4896* (+) | 13.78 | 13.60 | 27.13 | Positive | 13.16 | 13.98 | 26.79 | Positive |
| 2 | *5015* (+) | 14.26 | 14.66 | 26.60 | Positive | 13.16 | 13.73 | 27.10 | Positive |
| 3 | *5207* (+) | 16.46 | 16.33 | 30.67 | Positive | 14.9 | 17.56 | 29.76 | Positive |
| 4 | *5342* (+) | 16.53 | 16.45 | 28.95 | Positive | 16.06 | 17.21 | 31.58 | Positive |
| 5 | *5426* (+) | 16.68 | 16.28 | 30.62 | Positive | 16.22 | 20.3 | 29.25 | Positive |
| 6 | *4907* (+) | 17.31 | 17.95 | 28.18 | Positive | 16.59 | 17.83 | 27.58 | Positive |
| 7 | *5058* (+) | 18.98 | 18.82 | 26.88 | Positive | 19.33 | 35.79 | 27.02 | Positive |
| 8 | *5062* (+) | 23.89 | 24.00 | 25.90 | Positive | 22.88 | 23.79 | 25.42 | Positive |
| 9 | *5085* (+) | 23.91 | 23.89 | 27.48 | Positive | 22.29 | 22.96 | 27.62 | Positive |
| 10 | *5438* (+) | 25.01 | 25.61 | 31.28 | Positive | 24.4 | 25.52 | 30.70 | Positive |
| 11 | *4872* (+) | 26.61 | 26.81 | 31.19 | Positive | 25.04 | 27.07 | 30.88 | Positive |
| 12 | *4738* (+) | 28.24 | 28.30 | 27.54 | Positive | 28.2 | 29.43 | 29.88 | Positive |
| 13 | *5472* (+) | 30.15 | 30.86 | 29.62 | Positive | 28.91 | 29.99 | 29.09 | Positive |
| 14 | *4883* (+) | 30.44 | 30.87 | 28.25 | Positive | 30.35 | 31.97 | 28.89 | Positive |
| 15 | *5181* (+) | 30.86 | 30.29 | 29.54 | Positive | 28.99 | 29.29 | 30.66 | Positive |
| 16 | *4798* (+) | 31.98 | 31.19 | 27.92 | Positive | 31.43 | 34.43 | 27.50 | Positive |
| 17 | *5179* (+) | 34.07 | 34.60 | 31.58 | Positive | 33.29 | 34.23 | 30.51 | Positive |
| 18 | *4727* (+) | 34.18 | 33.60 | 31.14 | Positive | 32.2 | 31.96 | 31.95 | Positive |
| 19 | *4978* (+) | 34.92 | 35.16 | 28.83 | Positive | 32.3 | 32.62 | 27.29 | Positive |
| 20 | *5384* (+) | 35.58 | 34.26 | 26.10 | Positive | 33.21 | 32.77 | 26.86 | Positive |
| 21 | *2528* (+) | 35.84 | 35.04 | 26.10 | Positive | 34.45 | 35.36 | 26.40 | Positive |
| 22 | *5198* (+) | 35.99 | 35.21 | 31.66 | Positive | 31.75 | 31.59 | 30.72 | Positive |
| 23 | *5093* (+) | 36.04 | 35.55 | 26.03 | Positive | 33.15 | 33.99 | 26.11 | Positive |
| 24 | *5174* (+) | 36.11 | 35.62 | 25.57 | Positive | 33.82 | 32.42 | 26.44 | Positive |
| **25** | ***2514* (-)** | ND | ND | 26.36 | Negative | 35.03 | 37.91 | 26.42 | Positive |
| **26** | ***2529* (-)** | ND | ND | 27.40 | Negative | 35.2 | 34.66 | 25.32 | Positive |
| **27** | ***2486* (-)** | ND | ND | 28.96 | Negative | 36.62 | ND | 26.82 | Inconclusive |
| **28** | ***2513* (-)** | ND | ND | 27.26 | Negative | 34.47 | 35.15 | 26.63 | Positive |
| **29** | ***2489* (-)** | ND | ND | 25.33 | Negative | 36.38 | ND | 25.25 | Inconclusive |
| **30** | ***2487* (-)** | ND | ND | 27.44 | Negative | 37.17 | ND | 26.99 | Inconclusive |
| **31** | ***2477* (-)** | ND | ND | 28.85 | Negative | 35.34 | ND | 28.95 | Inconclusive |
| **32** | ***2473* (-)** | ND | ND | 25.90 | Negative | 37.32 | ND | 26.24 | Inconclusive |
| **33** | ***2517* (-)** | ND | ND | 28.32 | Negative | 37.32 | ND | 26.64 | Inconclusive |

Comparative Cq data for the TaqPath One-Step RT-qPCR kit and the dye-based open RT-qPCR reaction mix. Assigned sample number (# Sample) and clinical sample identifier (ID) are displayed. The clinical reports of the samples before they were re-tested by the two kits are also indicated in parentheses. The samples whose reports were altered are denoted in bold. **(-):** negative samples, **(+):** positive samples, **ND:** non-detected.
