# Supplementary material for: An Open One-Step RT-qPCR for SARS-CoV-2 detection
Source: PLoS One. 2024 Jan 25;19(1):e0297081. doi: 10.1371/journal.pone.0297081 (PMC10810446; doi:10.1371/journal.pone.0297081)
Supplement: S1 Raw images — (PDF) [file pone.0297081.s009.pdf]

Supplementary Figures

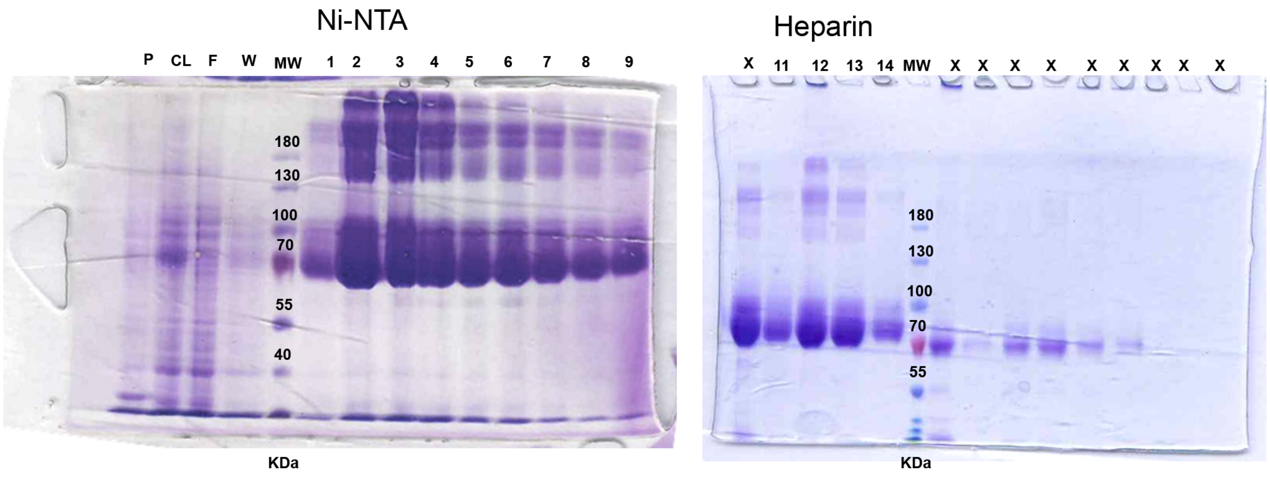

Supplemental Figure 1 (Panel A: M-MLV-RT) SDS-PAGE gels for Ni-NTA and Heparin. The images of the gels were obtained with a scanner.

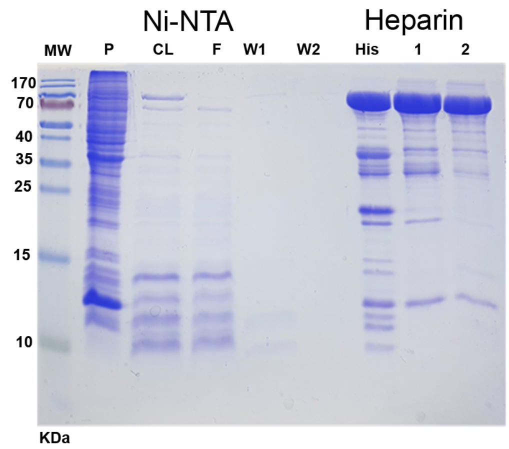

Supplemental Figure 1 (Panel B: Taq DNA Pol) SDS-PAGE gel for Ni-NTA and Heparin. The image was obtained with a scanner.

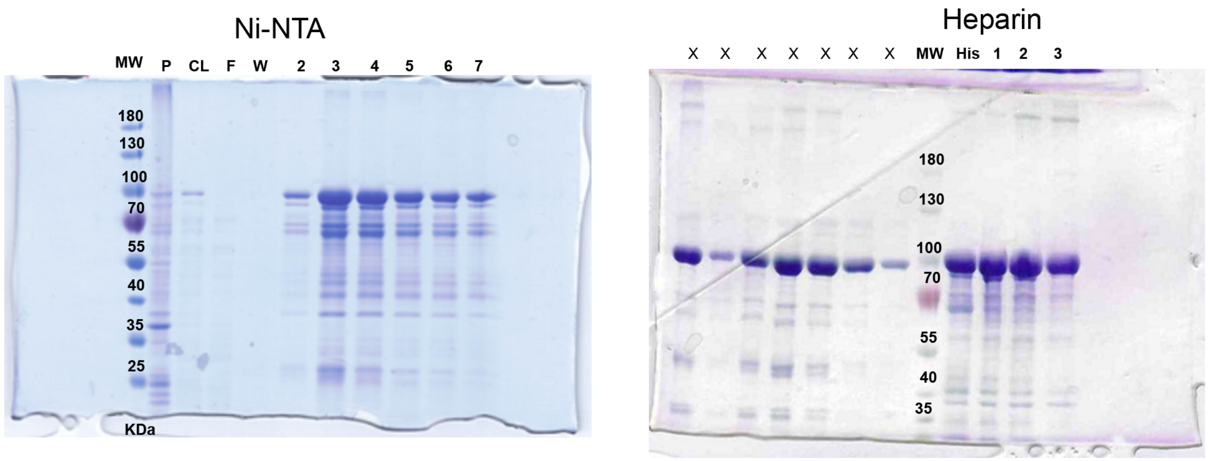

Supplemental Figure 1 (Panel C: Pfu-Sso7d) SDS-PAGE gels for Ni-NTA and Heparin. The images of the gels were obtained with a scanner.

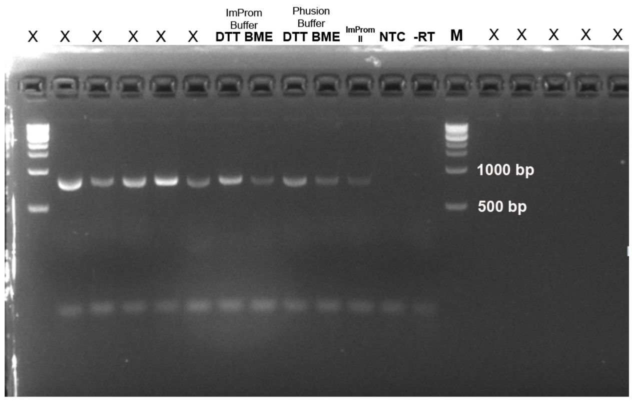

Supplemental Figure 2 (Panel A: Two-Step RT-PCR using M-MLV RT and Pfu-Sso7) Agarose gel pictures were obtained with an Imaging System.

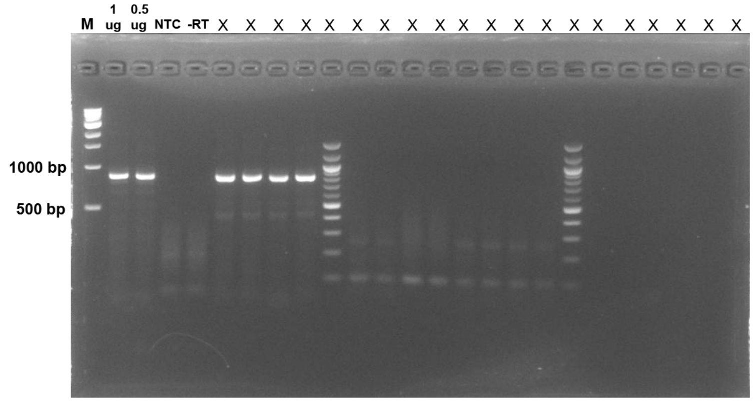

Supplemental Figure 2 (Panel B: One-Step RT-PCR using M-MLV RT and Taq DNA pol) Agarose gel pictures were obtained with an Imaging System.

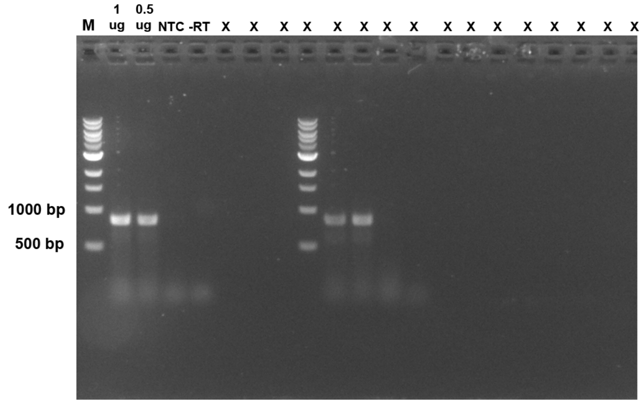

Supplemental Figure 2 (Panel C: One-Step RT-PCR using M-MLV RT and Pfu-Sso7d) Agarose gel pictures were obtained with an Imaging System.
