# Supplementary material for: An Open One-Step RT-qPCR for SARS-CoV-2 detection
Source: PLoS One. 2024 Jan 25;19(1):e0297081. doi: 10.1371/journal.pone.0297081 (PMC10810446; doi:10.1371/journal.pone.0297081)
Supplement: Attachment — Submitted filename: PONE-D-23-16856 Response to Reviewers.pdf [file pone.0297081.s011.pdf]

FACULTAD DE CIENCIAS BIOLÓGICAS
PONTIFICIA UNIVERSIDAD CATÓLICA DE CHILE

Rodrigo A. Gutiérrez, Ph.D.
Molecular Genetics and Microbiology
Facultad de Ciencias Biológicas
Pontificia Universidad Católica de Chile
Casilla 114-D, Santiago. Chile

Tel: +56 2 2686-2663
Fax: +56 2 2222-5515
E-mail: rgutierrez@bio.puc.cl

Santiago, December 18th, 2023

Haitham Mohamed Amer
Academic Editor
PLOS ONE

Dear Dr. Amer,

Please find enclosed the revised version of our manuscript entitled **"An Open One-Step RT-qPCR for SARS-CoV-2 detection"** [PONE-D-23-16856] - [EMID:486baa91abbf9866], by Ariel Cerda, Maira Rivera, Grace Armijo, Catalina Ibarra-Henriquez, Javiera Reyes, Paula Blázquez-Sánchez, Javiera Avilés, Aníbal Arce, Aldo Seguel, Alexander J. Brown, Yesseny Vásquez, Marcelo Cortez-San Martín, Francisco A. Cubillos, Patricia García, Marcela Ferres, César A. Ramírez-Sarmiento, Fernán Federici & Rodrigo A. Gutiérrez.

Thank you for the opportunity to revise our manuscript. We responded to the requirements requested by you as an editor and constructively addressed the points raised by the reviewer 3, as detailed below.

Reviewer #3: This a very important study, which describes the effectiveness of a homemade, probe-based One-Step RT-qPCR reaction mix in the detection of SARS-CoV-2 RNA in clinical samples. Furthermore, it proposed an economically viable production of homed-made M-MLV RT, Taq DNA pol and master mixes for clinical use.

Thank you for your positive comments and recognition of our work in developing a homemade One-Step RT-qPCR mix for SARS-CoV-2 detection. We believe this approach is vital for resource-limited settings and hope it significantly contributes to the scientific and medical communities. Your feedback has been invaluable in emphasizing the impact of our study.

In page 6, line 151 and 153; Page 7 165-167: Please include catalogue numbers of the kits used.

We have addressed your suggestion and updated the manuscript accordingly. The catalogue numbers for the kits have now been included in the specified sections (page 6, lines 151 and 153; page 7, lines 165-167).

In Page 7, line 163: The statement, "were collected from symptomatic cases of COVID-19 consultants at the UC-Christus Health" ……, is an incomplete sentence. Do you mean that samples were collected from consultants who were COVID-19 positive or, or from COVID-19 patients, or consultants attending to COVID-19 patients? You may wish to rephrase the sentence for easy comprehension.

We have amended the text to more precisely reflect that the samples were obtained from individuals who sought medical consultation at UC-Christus Health, displaying symptoms consistent with COVID-19. The modified sentence now reads: "On the other hand, 33 nasopharyngeal swabs in Universal Transport Medium (UTM) were collected from 17 to 22 July 2020 at the UC-Christus Health Network in Chile, from individuals presenting with symptoms suggestive of COVID-19." Additionally, we have included the following detail to provide further clarity: 'Of these, 24 were later confirmed as COVID-19 positive, while the remainder tested negative.' We believe this revision accurately depicts both the context of the patient consultations and the results of their COVID-19 tests. These same collected samples were analyzed using our dye-based RT-qPCR reaction mix for the purpose of comparing with the results obtained from clinical analyses.

The presentation of methodology and results are commendable. However, the authors did not describe the 'Limitation of the study'. What is/are the limitation(s) of the study? Can the findings be replicated in other independent laboratories outside of the home laboratory or home country? Please make 1-2 statements on study limitations.

Regarding limitations, we have added a paragraph to the discussion section acknowledging the challenges in replicating our findings in independent laboratories, particularly due to probe costs and the need for further standardization of our probe-free kit. We also acknowledge the relatively small sample size of our study. Furthermore, we highlight the role of collaborative networks like ReClone in facilitating reproducibility and global collaboration in similar assays.

Also, recommendation to include interlaboratory ring trials in some low to middle-income countries may be necessary (optional!)

We completely agree that this would greatly enhance the robustness and applicability of our findings. It aligns perfectly with our goal of establishing collaborative networks, which we are actively working on. We have added a section to our manuscript discussing this aspiration and its future potential. Due to logistical constraints at the time of this study, such trials were not feasible, but they are an important focus for our future research.

Please do not hesitate to contact us if you have any additional comments about this manuscript.

Sincerely yours,

Rodrigo A. Gutiérrez
Professor
Friedrich Wilhelm Bessel Scholar, Alexander von Humboldt Foundation
Deputy Director Millenium Institute for Integrative Biology
FONDAP Center for Genome Regulation

Molecular Genetics and Microbiology
Facultad de Ciencias Biológicas
P. Universidad Católica de Chile
